# Clinical characteristics and follow-up of complex arrhythmias associated with *RYR2* gene mutations in children

**DOI:** 10.3389/fgene.2024.1405437

**Published:** 2024-05-27

**Authors:** Yefeng Wang, Yufan Yang, Ningan Xu, Yunbin Xiao, Chao Zuo, Zhi Chen

**Affiliations:** ^1^ Department of Cardiology, The Affiliated Children’s Hospital of Xiangya School of Medicine, Central South University (Hunan Children’s Hospital), Changsha, China; ^2^ Department of Pediatric Intensive Care Unit, The Affiliated Children’s Hospital of Xiangya School of Medicine, Central South University (Hunan Children’s Hospital), Changsha, China; ^3^ Department of Children Health, The Affiliated Children’s Hospital of Xiangya School of Medicine, Central South University (Hunan Children’s Hospital), Changsha, China

**Keywords:** children, RyR2 gene, complex arrhythmias, atrial arrhythmias, gene mutation

## Abstract

**Objective:**

The aim of this study was to analyze the diagnosis, treatment, and follow-up of six cases of complex arrhythmias associated with *RYR2* gene mutations in children.

**Method:**

A retrospective analysis was conducted on six children diagnosed with complex arrhythmias associated with *RYR2* gene mutations. The study included an analysis of the age of onset, initial symptoms, electrocardiographic characteristics, genetic results, treatment course, and follow-up outcomes.

**Results:**

Among the six cases included in the study, there were four males and two females, with an average age of 3.5 ± 0.5 years. The average time from initial symptoms to diagnosis was 2.7 ± 1.3 years. The most common clinical manifestation was syncope, with exercise and emotions being the main triggers. All six children had *de novo* missense mutations in the *RYR2* gene identified through whole-exome sequencing. In Holter electrocardiogram, atrial arrhythmias and sinoatrial node dysfunction were commonly observed in younger children. Four patients underwent exercise stress testing, with two experiencing bidirectional ventricular premature contractions and two experiencing bidirectional ventricular tachycardia and polymorphic ventricular tachycardia. Initial treatment involved oral propranolol or metoprolol. If arrhythmias persisted, flecainide or propafenone was added as adjunctive therapy. Two patients received permanent cardiac pacemaker treatment (single chamber ventricular pacemaker, VVI). All patients survived, with three experiencing occasional syncope during treatment. The follow-up period ranged from 12 to 37 months, with an average follow-up time of 24.3 ± 3.7 months.

**Conclusion:**

Complex arrhythmias associated with *RYR2* gene mutations in children can present with various clinical manifestations. Atrial arrhythmias combined with sinoatrial node dysfunction are commonly observed in younger children, and the combination of pharmacological therapy and cardiac pacemaker treatment yields favourable treatment outcomes.

## 1 Introduction

The *RYR2* gene is a key pathogenic gene encoding a calcium ion release channel located in the sarcoplasmic reticulum of myocardial cells, which is an important component of myocardial excitation-contraction coupling (Pérez-Riera et al., 2018; Baltogiannis et al., 2019). Complex arrhythmia begins with a single abnormal complex that is progressing to grouped, sustained complexes associated with worsened symptoms and outcome. The clinical manifestations of complex arrhythmias related to *RYR2* gene mutations are varied, and electrocardiogram manifestations in children can vary, leading to potential misdiagnosis and missed diagnosis. In adult patients, most cases with *RYR2* gene mutations present as ventricular arrhythmias. But in children, it can develop various types of tachycardia (e.g., ectopic atrial tachycardia, atrial flutter) and sinus node dysfunction. This study aims to retrospectively analyze the clinical data and treatment follow-up of six children with complex arrhythmias associated with *RYR2* gene mutations, to provide evidence for early detection and rational treatment of this disease.

## 2 Patients and method

### 2.1 Patients

This was an observational retrospective, single-centre trial. Patients were enrolled between January 2017 and January 2023 at Hunan Children’s Hospital, Changsha, China. The definitions of complex arrhythmias associated with the *RYR2* gene are as follows: 1) Development of multiple types of arrhythmias. 2) The patient has *RYR2* gene mutations. This study protocol was approved by the Ethics Committee of the hospital (HCHLL-2023-128). All patients’ family members were aware that their clinical data might be used for a clinical study and signed written informed consent.

### 2.2 Method

Retrospective analysis of the clinical data of enrolled children was conducted, including medical history, physical examination, electrocardiogram, Holter ECG, cardiac ultrasound, and pathogenic gene analysis results. The exercise treadmill test (GET2100 exercise treadmill, United States) was performed with an improved Bruce regimen for submaximal exercise. The indications for termination of the exercise test were: 1) achieving the target heart rate [(220-age) x 85%]; 2) Malignant arrhythmias such as ventricular tachycardia appear; 3) Development of intolerable symptoms such as palpitations, dizziness, etc. Whole exome gene sequencing was performed by Guangzhou Jiajian Medical Laboratory.

The assessment of the pathogenic role of the genetic variants identified should be multiparametric. Judicious use of bioinformatic predictors, which are known for a high rate of false-positives, and *in vitro* studies where available, should be coupled with data derived from the families of the index case and population data. According to ACMG guidelines, we calculate the correlation using the following six scoring criteria: 1. Group frequency: total frequency of population variation. 2. Conservative analysis: the region where this mutation occurs is an important component of this protein, and the amino acid sequences of different species are highly conserved. 3. Algorithm prediction: Computer assisted analysis predicts a higher likelihood of this mutation affecting protein structure/function. 4. Public data. 5. Family analysis: parents carry *RYR2* gene mutation or not. 6. Clinical notes: Based on the clinical manifestations and family analysis of the examinee, the clinical correlation is strong. The score of case 1 is PM2-P + PP3-A1+PP3-B1+PS4-M + PP2+PM6. The score of case 2 is PM2-P + PP3-A1+PP3-B2+PM1+PP2+PM6. The score of case 3 is PM2-P + PP3-A1+PP3-B1+PM1+PP2+PM6. The score of case 4 is PM2-P + PP3-A1+PP3-S + PM1+PP2+PM6. The score of case 5 is PM2-P + PP3-A1+PP3-B1+PM5+PP2+PS2. The score of case 6 is PM2-P + PP3-A1+PP3-B1-M + PM5+PP2+PS2.

### 2.3 Treatment and follow-up procedure

All children were advised to limit their exercise and avoid emotional stimulation. They were started on β-receptor blocker therapy, initially with a dose of 0.5–1 mg/(kg·d) of propranolol, administered orally in three times, and gradually increased to a tolerable maximum dose of 2–4 mg/(kg·d). Alternatively, a starting dose of 0.5–1.0 mg/(kg·d) of metoprolol tartrate was administered orally in two times. If arrhythmias persisted despite the use of β-receptor blockers, oral flecainide (1 mg/kg, twice a day) or propafenone (5–7 mg/kg, three times a day) was added. If obvious sinus bradycardia occurred after medication treatment, implantation of a pacemaker was considered. Outpatient follow-up was conducted after discharge, with Holter ECG and echocardiography reviewed every 3–6 months to evaluate heart function and the presence of cardiovascular-related symptoms. Children over 5 years old were evaluated through exercise treadmill tests to clarify the heart rate limit for sinus tachycardia before arrhythmias occurred, with the aim of avoiding exceeding the threshold in daily life.

## 3 Results

### 3.1 Clinical characteristics

Among the six cases of complex arrhythmias associated with *RYR2* gene mutations, there were four boys and two girls. Three children were initially misdiagnosed as “epilepsy” and treated with antiepileptic drugs in other hospitals. Two children were misdiagnosed as “myocarditis and tachycardia cardiomyopathy”, and one child was misdiagnosed as “supraventricular tachycardia”. For three children who were misdiagnosed with epilepsy at the first diagnosis, their initial symptoms were syncope and convulsions. They were admitted to neurology department for the first time, and diagnosed with epilepsy according to their clinical symptoms and therefore they were misdiagnosed. Without epileptic wave in electroencephalogram (EEG), electrocardiogram (ECG) or exercise treadmill was performed to detect that they have complex arrhythmias. Finally, genetic test was performed to detect *RYR2* mutation and the diagnosis was corrected. For two children who were misdiagnosed with myocarditis and tachycardia cardiomyopathy at the first diagnosis. The onset of the disease was heart failure, with abnormal elevation of myocardial enzyme and cardiac troponin. And cardiac ultrasound showed cardiac enlargement. But later, they developed with complex arrhythmias such as atrial tachycardia and sinus node dysfunction. Finally, Genetic test was performed to confirm the diagnosis and found *RYR2* gene mutation. For two children who were misdiagnosed with supraventricular tachycardia at the first diagnosis. As the electrocardiogram diagnosis was supraventricular tachycardia, she developed with syncope and Holter ECG showed sinus arrest. So genetic testing was performed to confirm the diagnosis, revealing *RYR2* gene mutation. Three children developed syncope during exercise, one child during emotional arousal, and one child experienced transient loss of consciousness due to sinus bradycardia. The duration of syncope ranged from more than 10 s to a few minutes, with spontaneous recovery of consciousness. The age of initial symptoms ranged from 2.0 to 5.0 years, with an average age of 3.5 ± 0.5 years; the age at confirmed diagnosis ranged from 2.1 to 11 years, with an average age of 6.3 ± 1.4 years. The time from initial symptoms to diagnosis ranged from 0.1 to 8.4 years, with an average time of 2.7 ± 1.3 years.

### 3.2 Examinations

**FIGURE 1:**
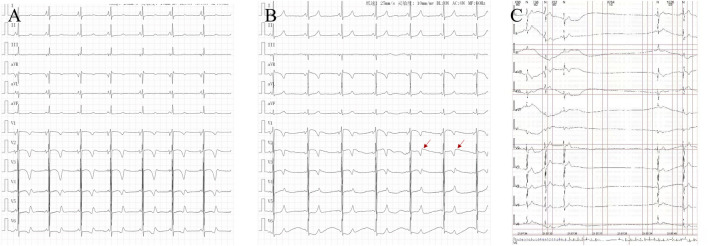
Characteristics of electrocardiogram in cases: **(A)**. Case 5: QT interval prolongation in electrocardiogram. **(B)**. Case 5: T-wave notches in lead V_2_ in electrocardiogram. **(C)**. Case 1: Holter ECG electrocardiogram recorded sinus arrest for 3.764 s.

**FIGURE 2:**
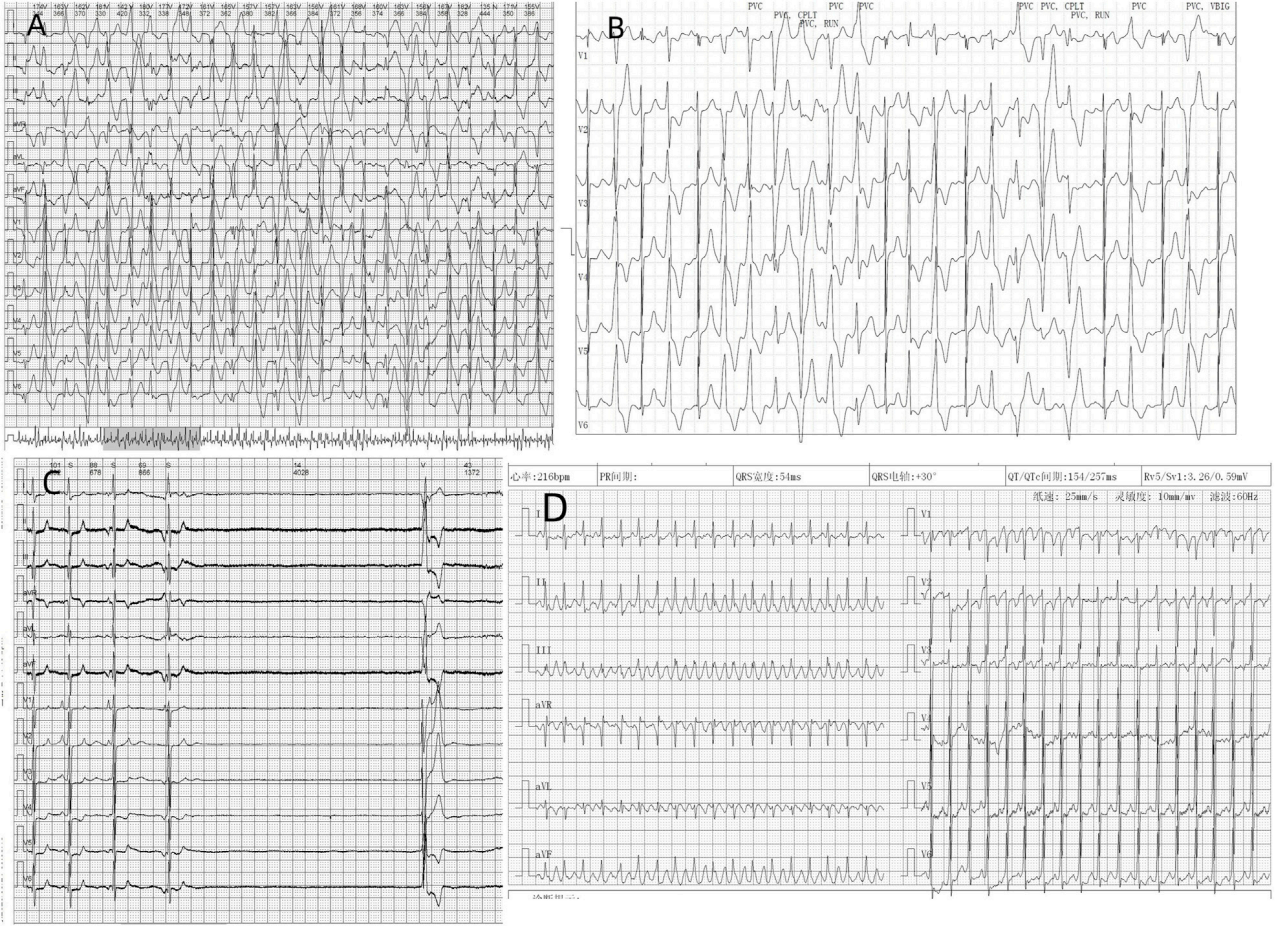
Different electrocardiogram manifestations in cases: **(A)**. Case 1 showed ventricular tachycardia recorded on dynamic electrocardiogram. **(B)**. Case 2: Ventricular premature and short paroxysmal ventricular tachycardia were recorded on the exercise treadmill. **(C)**. Case 3: Dynamic electrocardiogram recorded sinus arrest for 4.02 s. **(D)** Case 5: Routine electrocardiogram recording of atypical atrial flutter.

Two patients were admitted, and their routine surface electrocardiogram showed atrial tachycardia; one exhibited borderline tachycardia, one had occasional premature atrial contractions, and two patients had T-wave notches in lead V_2_. Six patients showed abnormalities in the Holter ECG, including four cases of atrial tachycardia, two cases of atrial flutter, one case of atrial fibrillation, three cases of sinus bradycardia and cardiac arrest (RR interval 2–4.4 s), and two cases of QT interval prolongation (QTc 476–545 m). One case developed short burst multi-source ventricular tachycardia, and three cases developed ventricular premature beats. The patients’ characteristics of electrocardiograms are shown in Figure 1 and Figure 2.

### 3.3 Genetic examination results

**FIGURE 3:**
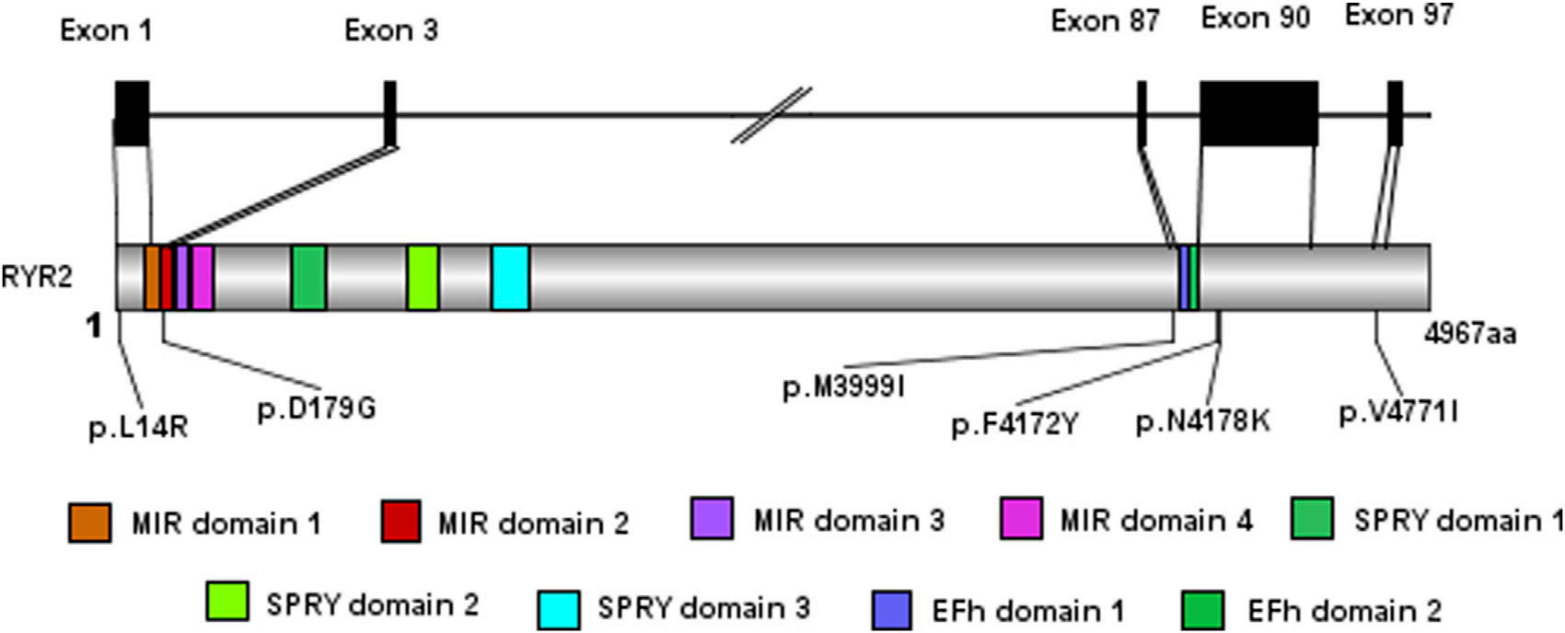
The model of exome gene sequencing and mapping results.

**FIGURE 4:**
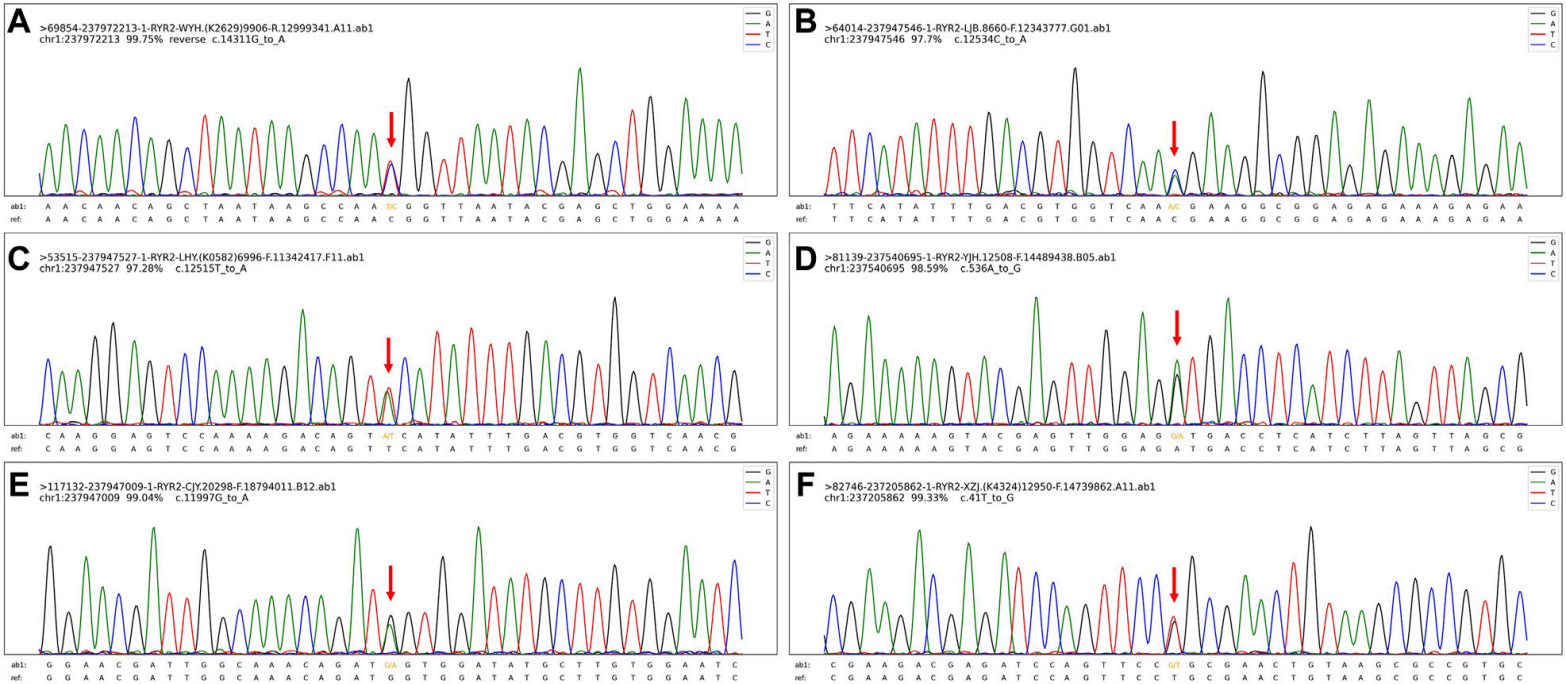
The exome gene sequencing and mapping results, especially for the mutation sites. **(A)**. The mutation site **(C)**.14311G>A (p.V4771I) in case 1. **(B)**. The mutation site **(C)**.12534C>A (p.N4178K) in case 2. **(C)**. The mutation site **(C)**.12515T>A (p.F4172Y) in case 3. **(D)**. The mutation site **(C)**.536A>G (p.D179G) in case 4. **(E)**. The mutation site **(C)**.11997G>A (p.M3999I) in case 5. **(F)**. The mutation site **(C)**.41T>G (p.L14R) in case 6.

All six cases underwent whole exome gene sequencing and parental verification, revealing *de novo* missense mutations, with no family history of related genetic diseases in the family members. The mutation site c.12534C>A (p.N4178K) in case 2 has not been reported in clinical cases, but other pathogenic mutations p.N4178S and p.N4178Y at the same amino acid position have been reported in clinical cases. The mutation site c.536A>G (p.D179G) in case 4 has not been reported in clinical cases, but the pathogenic mutation p.G178A near this amino acid position has been reported in clinical cases. The mutation site c.41T>G (p.L14R) in case 6 has not been reported in the literature, but the region of variation is an important component of this protein. The mutation site c.12515T>A (p.F4172Y) in case 3 has not been reported in the literature. Cases 1 and 5 have been reported in clinical cases (Ohno et al., 2015; Kaneshiro et al., 2017; Kannankeril et al., 2017; Wilde et al., 2022). Based on the clinical manifestations and test results of the examinees, and according to the American College of Medical Genetics and Genomics (ACMG) mutation classification guidelines, the pathogenicity of the mutation sites in all six children is possible. The genetic examination results are shown in Table 1. The gene test had been performed in their parents and no *RYR2* gene mutations were found in their parents. Their family members were unwilling to undergo the gene test. And the exome gene sequencing and mapping results, especially for the mutation sites, are shown in this paper (Figure 3; Figure 4).

**TABLE 1:** The results of gene test.

Patient	Gene	Heterozygous/homozygous	Nucleotide changes	Changes in amino acids	Source	Pathogenicity
1	RYR2	Heterozygous	c.14311G>A	p.V4771I	Old	Would be Pathogenicity
2	RYR2	Heterozygous	c.12534C>A	p.N4178K	New	Would be Pathogenicity
3	RYR2	Heterozygous	c.12515T>A	p.F4172Y	New	Would be Pathogenicity
4	RYR2	Heterozygous	c.536A>G	p.D179G	New	Would be Pathogenicity
5	RYR2	Heterozygous	c.11997G>A	p.M3999I	Old	Would be Pathogenicity
6	RYR2	Heterozygous	c.41T>G	p.L14R	New	Would be Pathogenicity

### 3.4 Treatment and follow-up

All six children were strictly restricted in exercise and received treatment with antiarrhythmic drugs. After oral administration of propranolol, two children did not develop syncope. Two patients still experienced intermittent palpitations and syncope after initial oral administration of metoprolol. However, after switching to propranolol, no palpitations or syncope occurred. One patient with atrial arrhythmias and frequent sinus arrest was treated with permanent epicardial pacing (VVI, VVI means a pacing mode: single chamber ventricular pacemaker), and after oral administration of propranolol and flecainide, the arrhythmias were significantly reduced. One patient developed significant sinus bradycardia after oral administration of metoprolol, and underwent permanent endocardial pacing (VVI, VVI means a pacing mode: single chamber ventricular pacemaker). One patient still experienced short paroxysmal ventricular tachycardia after taking propranolol orally, and propafenone was added orally. All cases survived, with a follow-up period of 12–37 months, with an average time of 24.3 ± 3.7 months. Please see Table 2 for details.

**TABLE 2:** Clinical characteristics of the patients.

Patient	Gender	Syncope	Holter ECG electrocardiogram	Treatment [mg/(Kg.d)]	Follow-up time (month)	Follow-up result
1	Female	Yes	atrial tachycardia, sinus arrest, Ventricular Tachycardia	Propranolol (1.5) propafenone (5)	37	Syncope for 2 times
2	Male	Yes	atrial tachycardia, ventricular premature, sinus bradycardia	Metoprolol(1), Later changed to Propranolol(2), VVI permanent pacing	28	Intermittent palpitations
3	Male	No	atrial tachycardia, atrial fibrillation, atrial flutter, sinus arrest	Propranolol (1.5), flecainide (2), VVI permanent pacing	30	Syncope for 1 time
4	Male	Yes	ventricular premature	Propranolol (2)	20	No discomfort
5	Female	No	atrial tachycardia, atrial flutter, sinus bradycardia, QT prolongation	Propranolol (2)	12	No discomfort
6	Male	Yes	junctional tachycardia, ventricular premature	Metoprolol(1), Later changed to Propranolol(2)	19	Syncope for 1 time

## 4 Discussion

Mutations in the *RYR2* gene are most commonly observed in patients with familial catecholaminergic polymorphic ventricular tachycardia (CPVT). Under catecholaminergic stimulation, these mutations can result in an excess Ca^2+^ load during diastole, leading to delayed afterdepolarization and subsequent arrhythmogenesis (Kim et al., 2020). In adult patients, most cases with *RYR2* gene mutations present as ventricular arrhythmias (Kawata et al., 2016). Faggioni et al. (Wilde et al., 2022) found that approximately 16% of patients with *RYR2* gene mutations develop various types of tachycardia (e.g., ectopic atrial tachycardia, atrial flutter) and sinus node dysfunction. The incidence rate of complex arrhythmias associated with *RYR2* gene mutations is low, and there are few cases reported globally. The early clinical manifestations are not typical, leading to insufficient understanding of the disease among pediatric clinicians and a propensity for missed diagnosis or misdiagnosis. In this series of cases, the age of initial symptoms ranged from 2.0 to 5.0 years, with all cases experiencing misdiagnosis during initial diagnosis. The time from initial symptoms to diagnosis ranged from 0.1 to 8.4 years (average time 2.7 ± 1.3 years). Older children are more likely to be misdiagnosed with epilepsy, while younger children are more likely to be misdiagnosed with paroxysmal supraventricular tachycardia, consistent with the reports of Al-Khatib et al. (Al-Khatib et al., 2018). In this series, four young children were found to develop various types of atrial arrhythmias (such as atrial fibrillation and atrial flutter) in their initial Holter ECG, accompanied by obvious sinus bradycardia or even sinus arrest. The arrhythmias were variable, accompanied by heart failure and enlargement, making diagnosis challenging. This study also discovered that in young children with *RYR2* gene mutations, the likelihood of developing atrial tachycardia is higher than that of ventricular tachycardia. As age increases, the probability of developing ventricular tachycardia and the probability of syncope also increase. Sinus node dysfunction and chronotropic dysfunction are also characteristic changes in this group of children. Dysfunction of calcium channels and voltage-gated channels plays an important role in the occurrence of sick sinus syndrome and has a significant influence on the sinus rhythm (Faggioni et al., 2014).

*RYR2* is a Ca^2+^ channel protein present in the sarcoplasmic reticulum of myocardial cells, playing a crucial role in regulating intracellular calcium ion flow and excitation-contraction coupling. Mutation in the *RYR2* gene can impair the function of the *RYR2* protein, leading to calcium leakage in the sarcoplasmic reticulum and causing fatal arrhythmias (Priori et al., 2013; Bongianino et al., 2017; Yamaguchi et al., 2022). To date, more than 350 mutations of *RYR2* have been reported, including splicing, deletion, insertion, and nonsense mutations, but most of them are missense mutations. It is challenging to clarify the association between mutation sites and pathogenicity, primarily due to the large size of the *RYR2* gene and the unclear relationship between numerous rare single nucleotide mutations and structural domains (Miyata et al., 2018). Currently, it has been found that 3.7% of the population carry benign *RYR2* gene mutations, making the interpretation of genetic results difficult. The expertise of professional genetic physicians is required to interpret the gene results (Steinberg et al., 2023). All cases in this study were newly diagnosed with missense mutations except case 1 and case 5, with mutation sites located in important regions of the *RYR2* domain. However, no family history of related genetic diseases was discovered in the family.

For the treatment of children with complex arrhythmias associated with *RYR2* mutations, attention should first be paid to lifestyle modifications, such as avoiding emotional excitement and competitive sports. β-receptor blockers are the first-line treatment drugs (Medeiros-Domingo et al., 2009). In this study, cases 2 and 6 still had symptoms even after taking metoprolol, but their condition improved significantly after switching to propranolol. Flecainide is an IC class antiarrhythmic drug that can inhibit delayed depolarization-mediated triggering activity by blocking sodium channels, as well as inhibit the release of sarcoplasmic reticulum calcium ions, reducing the occurrence of ventricular arrhythmias in these patients. In a randomized controlled study conducted in 2016 (Kannankeril et al., 2017), it was found that flecainide is more effective than β-receptor blockers in reducing the occurrence of ventricular tachycardia, although the number of patients in the study was small. In case 3, the occurrence of arrhythmia significantly decreased after the addition of flecainide.

For patients with sinus node dysfunction, increasing the rate of the sinus node can reduce the incidence of ventricular tachycardia and syncope. A study has found that using atropine to increase supraventricular heart rate can reduce the incidence of ventricular arrhythmias after exercise on a treadmill (Kannankeril et al., 2020). To avoid symptoms related to long R-R intervals occurring after β-blocker administration, patients can be administered β-receptor blockers under the protection of cardiac pacing therapy to reduce the occurrence of malignant arrhythmias (Writing Committee Members et al., 2021). In this study, cases 2 and 3 exhibited obvious symptoms related to long R-R intervals after receiving β-blockers. After receiving cardiac pacing treatment to increase the ventricular rate, no more malignant arrhythmias occurred, and good results were achieved.

We provide some potential advices on the diagnosis and treatment. 1. For children with syncope, while electroencephalogram were performed to exclude epilepsy, Holter ECG and exercise treadmill tests should be performed as early as possible. 2. When complex arrhythmias are detected by Holter ECG and exercise treadmill tests, genetic testing should be performed. 3. Timely and sufficient use of non-selective β Receptor blockers can control arrhythmia, avoiding emotional excitement and competitive exercise can significantly improve the prognosis of such pediatric patients. 4. For patients with sinus node dysfunction and tachycardia, adequate β receptor blockers administered under the protection of cardiac pacing can reduce the occurrence of malignant arrhythmias.

## 5 Study limitation

This is a single-centre study, and more valuable findings are likely to be uncovered in a nationwide, multicentre, large-sample study.

## 6 Conclusion

Complex arrhythmias associated with *RYR2* gene mutations in children can present with various clinical manifestations. Atrial arrhythmias combined with sinoatrial node dysfunction are commonly observed in younger children, and the combination of pharmacological therapy and cardiac pacing treatment yields favourable treatment outcomes.

## Data Availability

The original contributions presented in the study are included in the article/Supplementary material, further inquiries can be directed to the corresponding author.
